# Clathrin- and Caveolin-Independent Entry of Human Papillomavirus Type 16—Involvement of Tetraspanin-Enriched Microdomains (TEMs)

**DOI:** 10.1371/journal.pone.0003313

**Published:** 2008-10-02

**Authors:** Gilles Spoden, Kirsten Freitag, Matthias Husmann, Klaus Boller, Martin Sapp, Carsten Lambert, Luise Florin

**Affiliations:** 1 Institute for Medical Microbiology and Hygiene, University of Mainz, Mainz, Germany; 2 Paul Ehrlich Institute, Langen, Germany; 3 Department of Microbiology and Immunology, Feist-Weiller Cancer Center, Center for Molecular Tumor Virology, LSU Health Sciences Center, Shreveport, Louisiana, United States of America; Institut Pasteur Korea, Republic of Korea

## Abstract

**Background:**

Infectious entry of human papillomaviruses into their host cells is an important step in the viral life cycle. For cell binding these viruses use proteoglycans as initial attachment sites. Subsequent transfer to a secondary receptor molecule seems to be involved in virus uptake. Depending on the papillomavirus subtype, it has been reported that entry occurs by clathrin- or caveolin-mediated mechanisms. Regarding human papillomavirus type 16 (HPV16), the primary etiologic agent for development of cervical cancer, clathrin-mediated endocytosis was described as infectious entry pathway.

**Methodology/Principal Findings:**

Using immunofluorescence and infection studies we show in contrast to published data that infectious entry of HPV16 occurs in a clathrin- and caveolin-independent manner. Inhibition of clathrin- and caveolin/raft-dependent endocytic pathways by dominant-negative mutants and siRNA-mediated knockdown, as well as inhibition of dynamin function, did not impair infection. Rather, we provide evidence for involvement of tetraspanin-enriched microdomains (TEMs) in HPV16 endocytosis. Following cell attachment, HPV16 particles colocalized with the tetraspanins CD63 and CD151 on the cell surface. Notably, tetraspanin-specific antibodies and siRNA inhibited HPV16 cell entry and infection, confirming the importance of TEMs for infectious endocytosis of HPV16.

**Conclusions/Significance:**

Tetraspanins fulfill various roles in the life cycle of a number of important viral pathogens, including human immunodeficiency virus (HIV) and hepatitis C virus (HCV). However, their involvement in endocytosis of viral particles has not been proven. Our data indicate TEMs as a novel clathrin- and caveolin-independent invasion route for viral pathogens and especially HPV16.

## Introduction

Human papillomaviruses (HPVs) are nonenveloped viruses with a double-stranded circular DNA genome [1]. The icosahedral capsid contains 360 copies of the major structural protein L1 and a so far undefined number of the minor capsid protein L2 [2]. Over 100 different HPV types have been identified. Following infection of epithelial cells, they mainly cause benign epithelial warts on skin and mucosa. However, high-risk types, most often HPV16, are the primary etiologic agents for anogenital malignancies, in particular cervical cancer [1]. Host cell entry of HPV is initiated by binding of the virus particle to specifically modified heparan sulfate proteoglycans (HSPGs) [3], [4]. There is evidence that binding to HSPGs induces a conformational change in both capsid proteins, which is required for productive infection [5], [6]. Following binding, virus particles are taken up into the cell with slow kinetics. We have recently obtained first evidence for transfer of the virions to a secondary non-HSPG receptor molecule after conformational changes have occurred [7]. In addition to HSPGs, α6 integrin as well as laminin 5 have been suggested to function as transient receptors for HPV [8]–[10]. However, the entry mechanisms and the molecules involved are still a subject of much scientific debate. For HPV16, it was reported that entry occurs by clathrin-mediated endocytosis, whereas HPV31 was shown to use caveolar-mediated uptake mechanisms [11], [12].

In the present study we have readdressed the early mechanisms of HPV16 invasion into host cells following binding to HSPGs. In contrast to previous reports, we found no evidence for clathrin-mediated endocytosis. HPV16 entry and infection was also independent of caveolae- or lipid raft-mediated uptake mechanisms. Instead, we found a close association of virions with the tetraspanins CD63 and CD151 on the cell surface. HPV16 entry and infection of epithelial cells was inhibited using tetraspanin-specific antibodies or siRNA. All evidence indicates that tetraspanins are involved in HVP16 host cell entry.

Tetraspanins are an evolutionary conserved family of four transmembrane domain-containing proteins including at least 32 members in humans [13]. They are widely expressed in many cell types and tissues. One important feature of tetraspanins is their ability to interact laterally with each other and with other transmembrane proteins to form tetraspanin-enriched microdomains (TEMs), also called tetraspanin webs [14]. Within these webs tetraspanins control the activities of associated molecules. They modulate intercellular interactions including adhesion, migration, and synapse formation and are involved in the organization of membrane-signaling complexes. In addition, they are involved in intracellular protein transport as well as in endocytosis and exocytosis. The molecular basis for the broad functionality of tetraspanins appears to be the capacity to form multiple intermolecular interactions with a large but defined set of transmembrane and intracellular molecules. The molecular partners for various tetraspanins include proteoglycans, integrins, growth factor receptors, members of the immunoglobulin superfamily, complement-regulatory proteins, uroplakins, rhodopsin, and others [14]. Although TEMs are enriched in cholesterol they show a number of differences that distinguish them from conventional lipid rafts. While lipid rafts are disrupted following cholesterol depletion, TEMs are resistant. In addition, typical raft resident proteins, like GPI-anchored proteins and caveolin do not associate with tetraspanins [15].

Previous reports described various roles for tetraspanins in the life cycle of different viruses like human T-cell leukemia virus 1, canine distemper virus, and feline leukemia virus. More recently, the tetraspanin CD63 has been identified to be associated with sites of human immunodeficiency virus type 1 (HIV-1) assembly and may be incorporated into viral membranes [16]–[18]. Furthermore, the existence of specific TEMs in the plasma membrane is now well documented and it has been suggested that these microdomains can function as exit gateways for HIV-1 [19]. However, whether TEMs are also involved in endocytosis of viral particles is unclear. The potential impact of tetraspanins in the process of virus entry is suggested by the observation that CD63-specific antibodies or recombinant extracellular domains of specific tetraspanins can inhibit HIV infection [20], [21]. Similarly, CD81 has been implicated in the entry of HCV into its natural host cells [22].

## Results

### HPV16 virions on the cell surface and detection of uptake

To study early events of papillomavirus entry into human epithelial cells we employed HPV16 pseudovirions (PsVs) that have been widely used to analyze HPV biology and infection. PsVs were generated by expression of the capsid proteins L1 and L2 in cells harboring a reporter plasmid (GFP, YFP, or DsRed) that results in packaging of episomal DNA into L1/L2-capsids to produce infectious PsVs [23]. These PsVs were then either used in infection assays, where delivery of reporter plasmids into the nucleus was measured by expression of the fluorescent protein 48 hours post infection, or in entry assays where their invasion route was monitored by immunofluorescence deconvolution microscopy [24] using antibodies detecting the L1 capsid protein.

**Figure 1:**
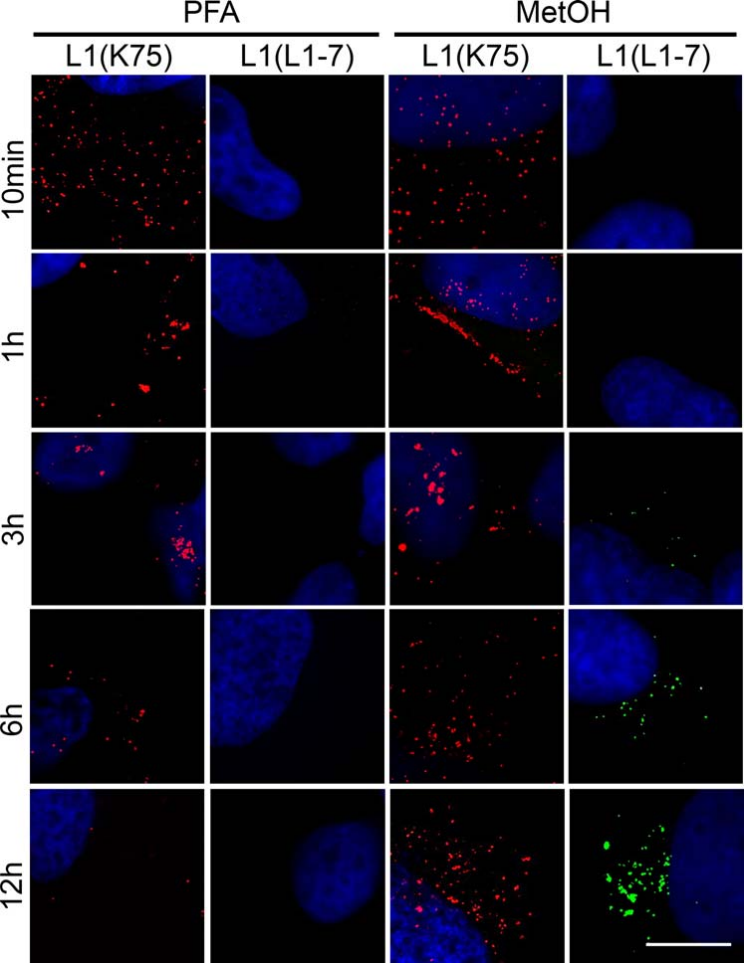
Differentiation between extra- and intracellular pseudovirions and kinetics of internalization. HeLa cells were exposed to HPV16 pseudovirions for the indicated time periods. Cells were fixed with paraformaldehyde (non-permeabilized cells, columns 1 and 2) or with methanol (permeabilized cells, columns 3 and 4). The major capsid protein L1 was detected with either a polyclonal anti-L1 antibody (K75, red) or with a monoclonal anti-L1 antibody (L1-7, green) reacting with an L1 epitope that is accessible exclusively after viral entry. Bar, 10 µm.

Two different approaches were used to differentiate whether virions were located at the cell surface or had been internalized. First, we compared non-permeabilized with permeabilized cells employing a polyclonal anti L1-antiserum (K75) that detects both extra- and intracellular particles. In order to exclusively analyze virions on the cell surface, cells were fixed with paraformaldehyde (PFA) leaving the plasma membrane impermeable for antibodies during labeling (Figure 1, PFA; Figure S1). Alternatively, cells were fixed and permeabilized with methanol (Figure 1, MetOH). Following the behavior of virions on the cell surface over time, we found pseudovirions at early stages after binding evenly distributed on the whole exterior of the cell (Figure 1, 10 min, K75, PFA and MetOH). At this stage, HPV16 is bound to proteoglycans, which act as primary attachment sites [7]. However, 1 hour after binding, virions were found to accumulate at more discrete areas at the cell surface (Figure 1, 1 h, 3 h, K75, PFA). In non-permeabilized cells the amount of particles on the cell surface decreased over time resulting in almost complete absence of K75 reactivity 12 hours after infection (Figure 1, 12 h, K75, PFA). The conclusion that this reflects entry of virions into the cell is supported by the detection of PsVs with the polyclonal antiserum in permeabilized cells at late time points after infection (Figure 1, K75, MetOH, 6 h–12 h).

In a second approach to follow entry of PsVs into the cell, we assessed the accessibility of a linear L1 epitope (amino acids 329–339) by monoclonal antibody (mAb) L1-7 [25]. The epitope recognized by this antibody is located in the interior of the PsV capsid and is not accessible for L1-7 in intact virions [26]. Therefore, pseudovirions located on the cell surface (non-permeabilized, PFA-treated cells) were detectable only with the L1-specific polyclonal antiserum K75 but not with mAb L1-7 at neither time point (Figure 1, PFA, L1-7; Figure S2, PFA). In agreement with the slow kinetics of papillomavirus uptake [5], [11], first L1-7 positive signals in permeabilized cells were detectable 3–4 hours after infection (Figure 1, L1-7, MetOH). The intracellular L1-7 signal increased over time, reflecting continuous viral entry (Figure 1, 3 h–12 h, L1-7, MetOH). As described, the amount of detectable PsVs on the plasma membrane concomitantly decreased (Figure 1, K75, PFA). Even 8 hours after infection, particles that were left on the cell surface were still exclusively detectable with the polyclonal antiserum (K75), but not with the L1-7 antibody (Figure S2, PFA). In contrast, both antibodies detected PsVs in the interior of permeabilized cells (Figure 1, 12 h, MetOH; Figure S2, PFA+Triton or MetOH), indicating that the L1-7 epitope is solely accessible in the cellular interior after entry.

### Endocytosis of HPV16 leading to productive infection is clathrin-independent

**Figure 2:**
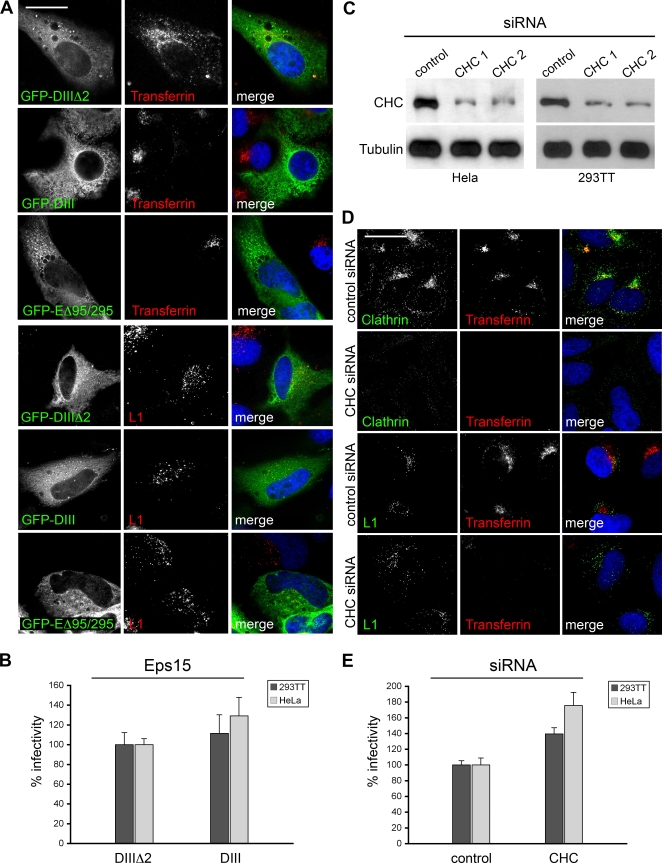
Dominant-negative inhibitors of clathrin-mediated endocytosis and knockdown of clathrin by siRNA do not influence infectious endocytosis of HPV16. (A) HeLa cells were transfected with GFP-tagged dominant-negative inhibitors of clathrin-mediated endocytosis or a control (GFP-DIIIΔ2). 24 hours after transfection cells were exposed to AlexaFluor 546 labeled transferrin or to HPV16 pseudovirions for 10 hours and then fixed with methanol. L1 was detected with L1-7. Bar, 20 µm. (B) 293TT (dark gray) and HeLa cells (light gray) were transfected with GFP-tagged dominant-negative inhibitor of clathrin-mediated endocytosis (DIII) or a control (GFP-DIIIΔ2) for 24 hours and then infected with HPV 16 PsVs. Data is shown as mean of infected cells within the GFP-positive cell population (n = 4, +/−SD); infection rate of the control (GFP-DIIIΔ2) was set to 100%. (C) siRNA mediated knockdown of clathrin in HeLa and 293TT cells by two different clathrin heavy chain (CHC) siRNAs was controlled 48 hours after transfection by Western blotting. (D) siRNA transfected HeLa cells were exposed to pseudovirions for 10 hours and/or AlexaFluor 546 labeled transferrin. Cells were fixed with methanol, immunostained with L1-7 or clathrin antibody and analyzed by immunofluorescence microscopy. All exposures were taken with identical settings. Bar, 20 µm. (E) Infection assay was performed in clathrin or control siRNA transfected cells (n = 4, +/−SD); infection rate of the control was set to 100%.

It has been reported that HPV16 enters target cells by clathrin-mediated endocytosis [11], [12]. This conclusion was mainly based on experiments using chlorpromazine, an inhibitor of clathrin-dependent endocytosis, which exerts multiple side effects on cell function as it targets many receptors, intracellular enzymes, and alters plasma membrane characteristics [27]. Therefore, we readdressed the question if entry of HPV16 is mediated by clathrin-dependent endocytosis employing more specific assays. First, we tested whether entry of HPV16 pseudovirions into HeLa cells was affected by the expression of dominant-negative (dn) Eps15 mutants. Eps15 is a component of clathrin-coated pits where it interacts with adaptor protein (AP)-2, the major clathrin adaptor complex, and overexpression of dnEps15 mutants has been shown to inhibit clathrin-dependent endocytosis [28]. Transferrin, a bona fide cargo of clathrin-coated pits served as control. We used two different dnEps15 mutants and one control mutant, which are all fused to GFP. One mutant contains a partial deletion of the DI-domain (EΔ95/295), whereas the other mutant has a large N-terminal deletion leaving only its C-terminal DIII-domain. To control for unspecific effects by overexpression, the DIII-mutant with an additional deletion of its AP-2 binding sites (DIIIΔ2) was used. In line with previous reports, overexpression of both dn-mutants efficiently blocked clathrin-dependent uptake of transferrin (Figure 2A, rows 2 and 3) whereas the control construct GFP- DIIIΔ2 had no effect on transferrin internalization (row 1). In contrast, entry of HPV16, which was monitored by the accessibility of L1-7 epitope (see Figure 1), was not influenced by the dnEps15-mutants (Figure 2A, rows 4–6).

To assess whether inhibition of clathrin-mediated endocytosis affected infection, 293TT and HeLa cells were first transfected with dn- or control-Eps15 mutants and then incubated with PsVs carrying a DsRed marker plasmid. Expression of the different Eps15 mutants was detected by GFP fluorescence (Figure S3, left column). Infected cells expressed DsRed (Figure S3, middle column). Importantly, a significant portion of cells expressing the dn Eps15 mutant were efficiently infected as indicated by yellow fluorescence (Figure S3, right column). In order to quantify the efficiency of HPV16 infection in cells treated with dnEps15-mutants we measured the number of transfected cells expressing the DsRed-marker plasmid by FACS analysis. In comparison to control cells expressing GFP-DIIIΔ2, we found no inhibitory effect on pseudovirus infection by dnEps15 GFP-DIII mutant (Figure 2B). In HeLa cells we even observed a slight increase of infectivity.

The main components of clathrin-coated pits and vesicles are clathrin triskelions, consisting of three heavy and three light chains. The clathrin lattice serves as an organizing scaffold for the proteins that carry out cargo sorting, membrane invagination, vesicle scission, and uncoating. siRNA-mediated depletion of the clathrin heavy chain (CHC) has been shown to efficiently block clathrin-mediated endocytosis [29]. Therefore, we also used knockdown of CHC (Figure 2C) to analyze whether entry of HPV16 into HeLa cells is dependent on clathrin-mediated endocytosis. Cells were either transfected with control siRNA or CHC-specific siRNA followed by incubation with PsVs or transferrin and analyzed by fluorescence microscopy. As shown in figure 2C and 2D, siRNA treatment substantially reduced the level of CHC (Figure 2D, compare left panels in row 1 and 2) leading to a complete inhibition of clathrin-mediated transferrin uptake (compare middle panels in row 1 and 2). However, entry of HPV16 was not affected by the inhibition of clathrin-mediated endocytosis as shown by analyses using the monoclonal antibody L1-7 (Figure 2D, row 4). These results were reproduced with the second CHC-specific siRNA (CHC2, data not shown).

Again, the findings obtained by immunofluorescence microscopy were confirmed by infection assays in siRNA-treated cells. The specific knockdown of CHC was controlled by Western blot analysis (Figure 2C) and infected cells were counted by FACS. As shown in figure 2E, we found no inhibitory effect on HVP16 infection of 293TT and HeLa cells when CHC was depleted. In fact, we detected increased infectivity. Altogether, these data indicated that HVP16 entry into epithelial cells for productive infection is not mediated by a clathrin-dependent mechanism.

### Infection of HPV16 does not occur via lipid rafts or caveolae

**Figure 3:**
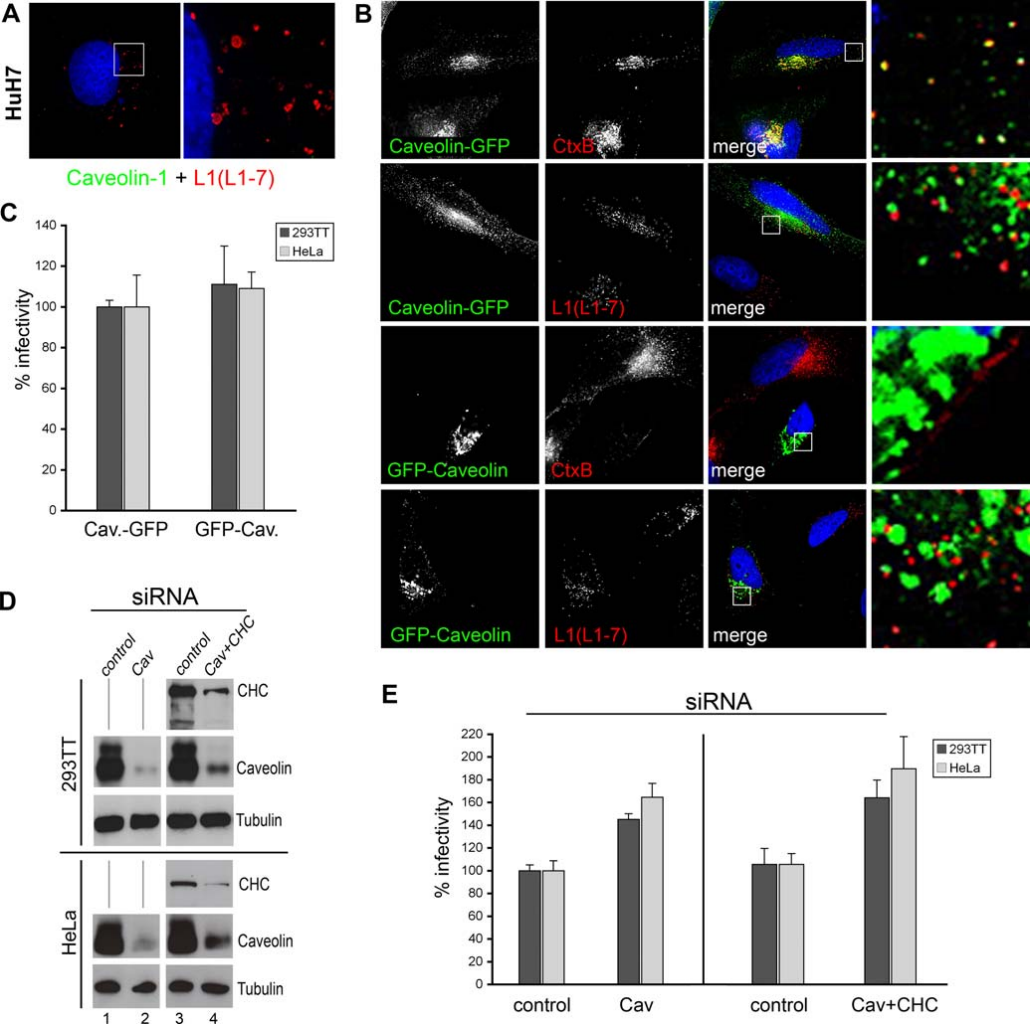
Infection of HPV16 does not occur via lipid rafts or caveolae. (A) HuH7 liver cells were incubated with HPV16 pseudovirions for 6 hours and fixed with methanol. Cells were immunostained with monoclonal anti-L1 antibody (L1-7, red) and with polyclonal anti caveolin-1 antibody (green). Insert display the enlarged section that is shown on the right. (B) HeLa cells were transfected with Caveolin-GFP or dominant-negative GFP-Caveolin as indicated and treated with 0.5 µg/ml AlexaFluor 594 labeled Cholera toxin B (CtxB) for 30 min or exposed to HPV16 pseudovirions for 10 hours. Cells were fixed with methanol and immunostained with L1-7 antibody as indicated. Bars in A and B, 20 µm. (C) 293TT (dark gray) and HeLa (light gray) cells expressing caveolin constructs as indicated for 24 hours were exposed to HPV16 pseudovirions and the number of infected cells was measured by FACS 48 hours post infection. Data of four individual experiments are represented as mean+/−SD. Infection rate of the control Caveolin-GFP was set to 100%. (D) 293TT or HeLa cells were transfected with caveolin-1 (Cav, lane 2) or a mixture of caveolin-1 and clathrin heavy chain (CHC) specific siRNAs (Cav+CHC, lane 4), or control siRNA (lanes 1 and 3). 48 hours after transfection, cells were lysed and probed for Caveolin and CHC by Western blotting or infected with HPV16 PsVs (E). Infectivity was quantified by FACS (n = 4, +/−SD); infection rate of the control was set to 100%.

HPV31 has been recently suggested to enter human keratinocytes via caveolae-dependent endocytosis [12]. Therefore, it was of interest to determine whether HPV16 also uses caveolae for entry into HeLa cells. Since the human hepatoma cells (HuH-7) express only extremely low levels of caveolins and are devoid of morphologically identifiable caveolae [30], we used these cells as a tool in entry experiments with HPV16. As shown in figure 3A, HPV16 PsVs efficiently entered these caveolae-deficient cells. To block caveolar-mediated endocytosis in HeLa cells, we expressed two GFP-tagged versions of caveolin-1: a construct with C-terminally fused GFP (Cav-GFP) that was shown to function as a marker for caveolae, and an N-terminally tagged version (GFP-Cav) that acts as dominant negative mutant inhibiting caveolar-mediated endocytosis [31]. Cholera toxin subunit B (CtxB), which enters cells mainly by a caveolar-mediated entry route when applied at low concentrations, was used as a marker [32], [33]. We confirmed a strong colocalization of Cav-GFP and CtxB (Figure 3B, row 1). CtxB endocytosis was inhibited in cells expressing the dominant negative GFP-Cav mutant (Figure 3B, row 3). However, HPV16 entry was not prevented under these conditions, as indicated by intracellular detection of virions with mAb L1-7 (Figure 3B rows 2 and 4). Cav-GFP and virions did not colocalize inside the cell. Moreover, no colocalization was found using the polyclonal L1-antiserum (K75) at any time point after infection (data not shown). Finally, we tested the dominant negative GFP-Cav mutant in infection assays and found no influence on infectivity (Figure 3C). In addition, disruption of lipid rafts and caveolae by depletion of cholesterol from cellular membranes using methyl-β-cyclodextrin (MβCD) did not impair infection, rather led to increased infectivity (data not shown). Furthermore, we depleted caveolin-1 by siRNA treatment and tested the effect on HPV16 infection. The specific knockdown of caveolin-1 in 293TT and HeLa cells was controlled by Western blot analysis (Figure 3D, lanes 1 and 2) and infectivity was measured by FACS (Figure 3E). As shown, HVP16 infection was slightly increased in caveolin-1-depleted cells. As it is conceivable that HPV16 could switch to another invasion pathway, we depleted clathrin and caveolin-1 simultaneously (Figure 3D, lanes 3 and 4). Intriguingly, infection efficiency of HPV16 PsVs was increased more than 50% when both clathrin and caveolin-1 mediated entry pathways were blocked. In sum, our data provided no evidence for the involvement of caveolae in the entry process of HPV16.

### Dynamin-independent entry of HPV16

**Figure 4:**
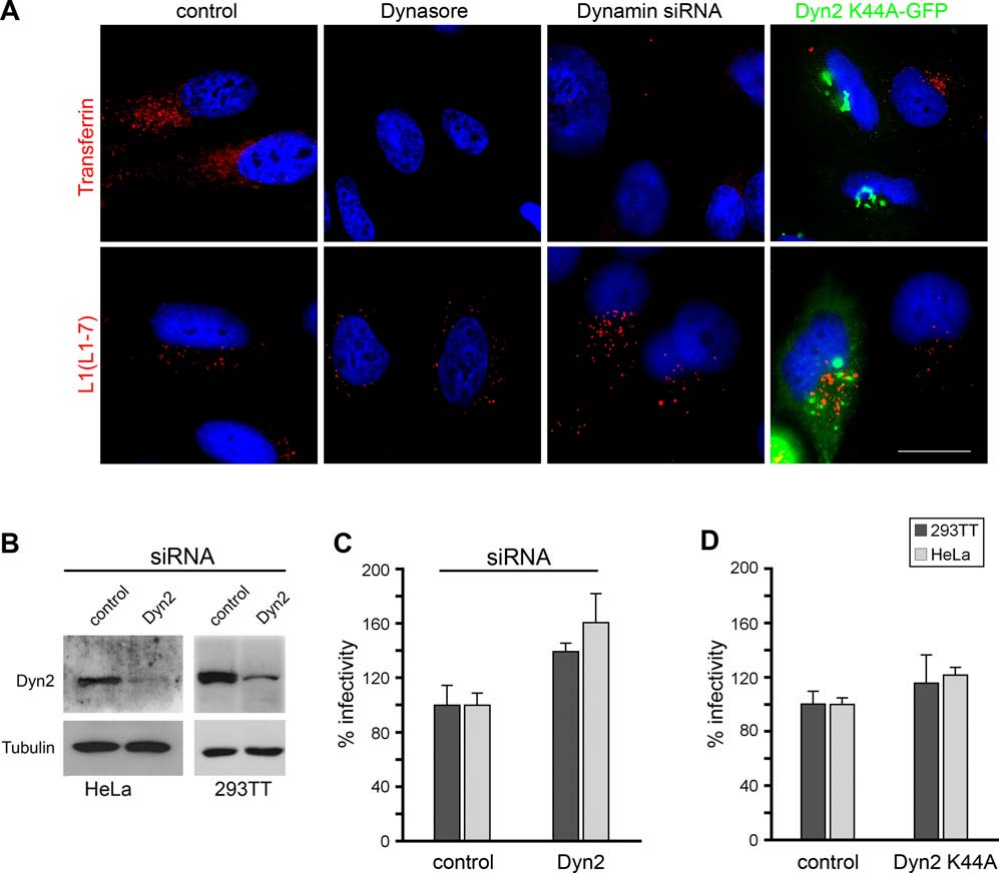
Dynamin-independent entry and infection of HPV16 PsVs. (A) HeLa cells were either mock treated (control), treated with 80 µM dynasore for 30 min, transfected with dynamin2 specific siRNA for 48 hours (Dynamin siRNA), or transfected with GFP-tagged dynamin2 K44A mutant for 24 hours (Dyn2 K44A-GFP). Thereafter, cells were incubated with AlexaFluor546 labeled transferrin or infected with HPV16 pseudovirions for 6 hours (dynasore: 4 hours). Cells were fixed and permeabilized with methanol. Immunostaining was performed with the indicated L1-specific antibody (L1-7, red). Cells were examined by immunofluorescence microscopy. Bar, 20 µm. (B) siRNA mediated knockdown of dynamin2 (Dyn2) in HeLa and 293TT cells was controlled 48 hours after transfection by Western blotting. (C, D) Infection assay was performed in dynamin2 or control siRNA transfected as well as in dn dynamin2 mutant (Dyn2 K44A) or control transfected 293TT (dark gray) and HeLa (light gray) cells. Infectivity was quantified by FACS (n = 4, +/−SD); infection rate of the control was set to 100%.

As it has been shown that the internalization of clathrin-coated vesicles and the uptake of caveolar microdomains are dependent on the functionality of the large GTPase dynamin we additionally analyzed the impact of dynamin for HPV16 entry and infection. In a first approach we inhibited dynamin function by treatment of HeLa cells with the inhibitor dynasore [34]. Whereas uptake of transferrin was efficiently inhibited (Figure 4A, Dynasore, upper panel), we found effective entry of HPV16 PsVs as indicated by the reactivity of the monoclonal antibody L1-7 (Figure 4A, Dynasore, lower panel). The same results were obtained using depletion of dynamin-2 by specific siRNA treatment (Figure 4A, Dynamin siRNA). The effectiveness of dynamin-2 depletion in HeLa cells was controlled by Western blot analysis (Figure 4B). In a third approach we specifically blocked the functionality of dynamin-2 by expression of the GPF-tagged dominant negative GTPase-deficient mutant Dyn2K44A-GFP [35]–[37]. Again, endocytosis of transferrin was inhibited in cells expressing the dominant-negative mutant, whereas transferrin was efficiently taken up in non-transfected cells (Figure 4A, Dyn2K44A-GFP, upper panel). However, entry of virions was not affected by expression of Dyn2K44A (Figure 4B, Dyn2K44A-GFP, lower panel). In order to test whether this dynamin-independent entry of virions results in productive infection, we performed infection assays in 293TT and HeLa cells. As shown in figure 4C, we found no inhibitory effect on infection efficiency when dynamin-2 was depleted with siRNA, or when we expressed the dn Dyn2K44A-mutant (Figure 4D). Rather, in any case we repeatedly observed a slight increase in the number of infected cells. These data confirmed our observations that entry and infection of HPV16 occur independently of clathrin- and caveolin-mediated endocytosis, since both pathways require the function of the large GTPase dynamin.

### Virions associate with tetraspanin-enriched microdomains during infection

**Figure 5:**
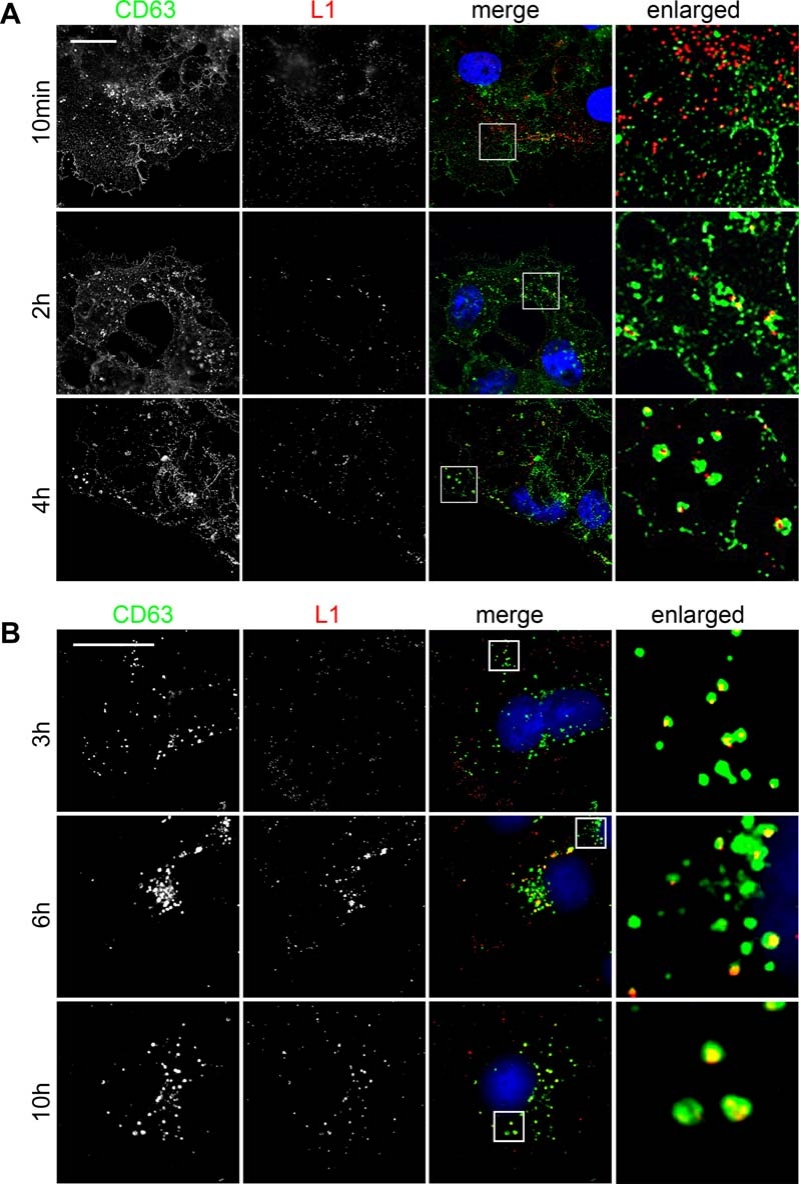
Virions associate with CD63 positive microdomains on the cell surface and in intracellular vesicles. (A) PsVs on the cell surface. HeLa cells were exposed to HPV16 pseudovirions for the indicated time periods, and fixed with paraformaldehyde. Cells were immunostained with polyclonal anti-L1 antibody (K75, red) and a monoclonal anti-CD63 antibody (H5C6). Inserts display enlarged sections that are shown in the right column and DNA staining in blue (A and B). (B) Treatment of HeLa cells with HPV16 pseudovirions as in A. Cells were fixed with methanol, immunostained with a polyclonal anti-L1 antibody (K75, red) and a monoclonal anti-CD63 antibody (sc-5275) recognizing only internal CD63 under these conditions, and analyzed as in A. Bars, 20 µm.

**Figure 6:**
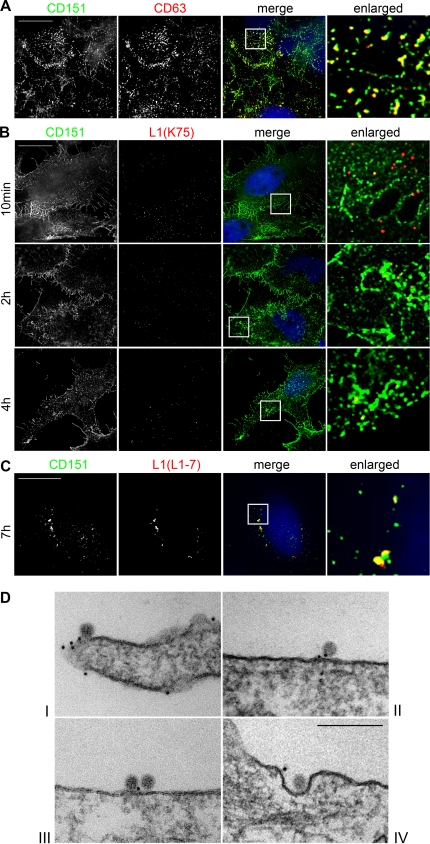
Virions associate with CD151 positive microdomains on the cell surface and in intracellular vesicles. (A) HeLa cells were fixed with paraformaldehyde and the cell surface was immunostained with a monoclonal anti-CD63 antibody and polyclonal anti-CD151 antibody. (B) HeLa cells were exposed to HPV16 pseudovirions for the indicated time periods, and fixed with paraformaldehyde. The cell surface was immunostained with polyclonal anti-L1 antibody (K75, red) and a monoclonal anti-CD151 antibody (sc-5275). Inserts display enlarged sections that are shown in the right column. (C) HeLa cells were exposed to HPV16 pseudovirions for 7 hours. Cells were fixed and permeabilized with methanol. Intracellular PsVs were immunostained with monoclonal anti-L1 antibody (L1-7, red) and CD151 with a polyclonal anti-CD151 antibody (sc-33123, green). Bars, 20 µm. (D) HeLa cells were infected with pseudovirions for 4 hours and then fixed with paraformaldehyde. Cell surface CD151 was immunolabeled with 10 nm gold particles. Pseudovirions and CD151 were visualized by electron microscopy. Bar, 200 nm.

Tetraspanins are concentrated at distinct sites in the plasma membrane, where they contribute to the formation of TEMs [19]. To detect surface localized TEMs, PFA-fixation was employed without permeabilizing the plasma membrane. As described by Nydegger and colleagues, the tetraspanin CD63 was found to be associated with discrete microdomains that were distributed over the cell surface (Figure S4, top section) or concentrated at the cell borders (Figure S4, middle section), depending on the focusing plane. When cells were permeabilized with methanol, surface staining got lost but perinuclear CD63-positive endosomes were detected (Figure S4, MetOH). We employed co-staining of virions and CD63 to follow the association of PsVs with CD63-positive TEMs over time. Using paraformaldehyde-fixed non-permeabilized cells we found virions evenly distributed on the cell surface and only marginal colocalization with plasma membrane CD63 was observed (Figure 5A, 10 min). However, colocalization gradually increased over time (2 h, 4 h). We found that virus particles started to colocalize with internal CD63 at the cell periphery already 3 hours after infection. PsVs appeared to be transported to CD63-positive endosomes near the nucleus (Figure 5B, 6 h and 10 h).

Next we analyzed the association of virions with another member of the tetraspanin family. The tetraspanin CD151 is highly abundant in the basolateral surface of basal keratinocytes [38]. Since papillomaviruses are believed to enter basal keratinocytes via the basolateral surface [39], we sought to determine whether CD151 was also located in TEMs on the plasma membrane and whether it played a role in HPV16 PsV entry. We first analyzed its surface distribution in HeLa cells and found strong colocalization with the TEM-marker CD63 (Figure 6A). When we studied the localization of pseudovirions relative to CD151, we again found almost no colocalization on the plasma membrane at early stages of infection (Figure 6B, 10 min). Quantitative analysis revealed that 10 minutes after addition of pseudovirions only 4±3% of particles per cell were colocalized with CD151. However, as seen with CD63, we detected increasing colocalization of HPV16 particles with CD151 on the cell surface as the infection process proceeded. Measurement of colocalization revealed that after one hour 9±1%, after two hours 20±4%, and after four hours 36±4% of PsVs were associated with CD151 positive TEMs on the cell surface. The association with CD151 was also sustained in intracellular endosomes near the nucleus later in infection (Figure 6C).

To substantiate these results we analyzed the association of virions with plasma membrane CD151 by electron microscopy. HeLa cells were grown on a film base and infected with HPV16 PsVs for 4 hours. To detect CD151 at the plasma membrane we performed preembedding immunogold labeling of non-permeabilized formaldehyde fixed cells. The monolayer of cells on the film base was embedded in epoxy resin and cut into ultrathin sections allowing the preservation of virions in the context of their microenvironment. As shown in figure 6D HPV16 PsVs colocalized with CD151 on the plasma membrane (I–III). Association with CD151 was also maintained during membrane invagination (IV). We did never observe virions in clathrin-coated pits. Altogether, these results supported the notion that HPV16 associates with tetraspanin proteins on the plasma membrane and implicated that TEMs might act as entry platforms for clathrin- and caveolin-independent entry of HPV16.

### Antibodies and siRNA specific for different tetraspanins can block HPV16-infection

**Figure 7:**
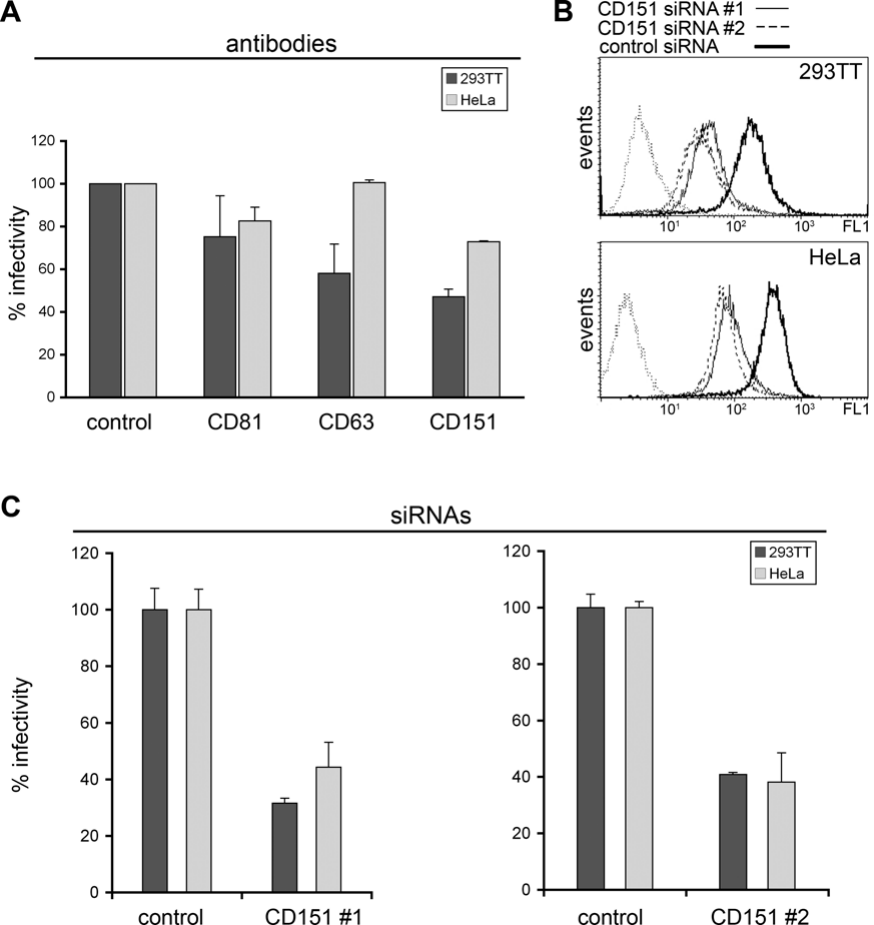
Tetraspanin specific antibodies and siRNA can block HPV16 infection. (A) 293TT and HeLa cells were preincubated with control antibody (rabbit IgG) or with tetraspanin specific antibodies as indicated. One hour later infection assay was performed (n = 4, +/−SD); infection rate of the control was set to 100%. (B) Flow cytometry analysis of the siRNA mediated knockdown of cell surface exposed tetraspanin CD151 in 293TT (upper panel) and HeLa (lower panel) cells. Dotted line represents control staining without primary antibody. (C) 293TT and HeLa cells were transfected with siRNA as indicated for 48 hours and than infection assay was performed (n = 4, +/−SD); infection rate of the control siRNA was set to 100%.

As we had detected HPV16 virions associated with TEMs on the plasma membrane, we asked whether tetraspanins are functionally involved in entry and infection. Accordingly, we first tested if pretreatment of 293TT and HeLa cells with tetraspanin-specific antibodies might inhibit infection. Three antibodies specifically recognizing the extracellular domains of CD63, CD151, and CD81 were used. As shown in figure 7A, anti-CD81 had some inhibitory effect on HPV16 infection in 293TT (25±19%) and HeLa (18±6%) cells. Antibodies against CD63 had a strong inhibitory effect in 293TT cells (42±13% inhibition) but showed no reduction of infection in HeLa cells. The strongest reduction of infection in both cell lines was found with anti-CD151. These antibodies reduced infectivity to about 47±3% in 293TT and 72±1% in HeLa cells.

Since antibody-inhibition studies suggested that the tetraspanin CD151 is primarily involved in HPV16 infection, siRNA treatment was used to reduce the amount of cell surface-exposed CD151. Efficiency of siRNA-mediated knockdown of CD151 on the cell surface was controlled by FACS analysis. As shown in figure 7B, siRNA #1 reduced cell surface expression of CD151 to 29% in 293TT and 26% in HeLa cells (mean intensity). siRNA #2 treatment had a similar efficiency with reduction of CD151 expression on the cell surface to 22% in 293TT and 23% in HeLa cells, as compared to control treated cells. Importantly, with CD151-specific knockdown, infectivity was markedly reduced in both cell lines. CD151-specific siRNAs reduced infectivity to 31±2% (siRNA #1) or 41±2% (siRNA #2) in 293TT and 42±8% (siRNA #1) or 38±10% (siRNA #2) in HeLa cells (Figure 7C).

**Figure 8:**
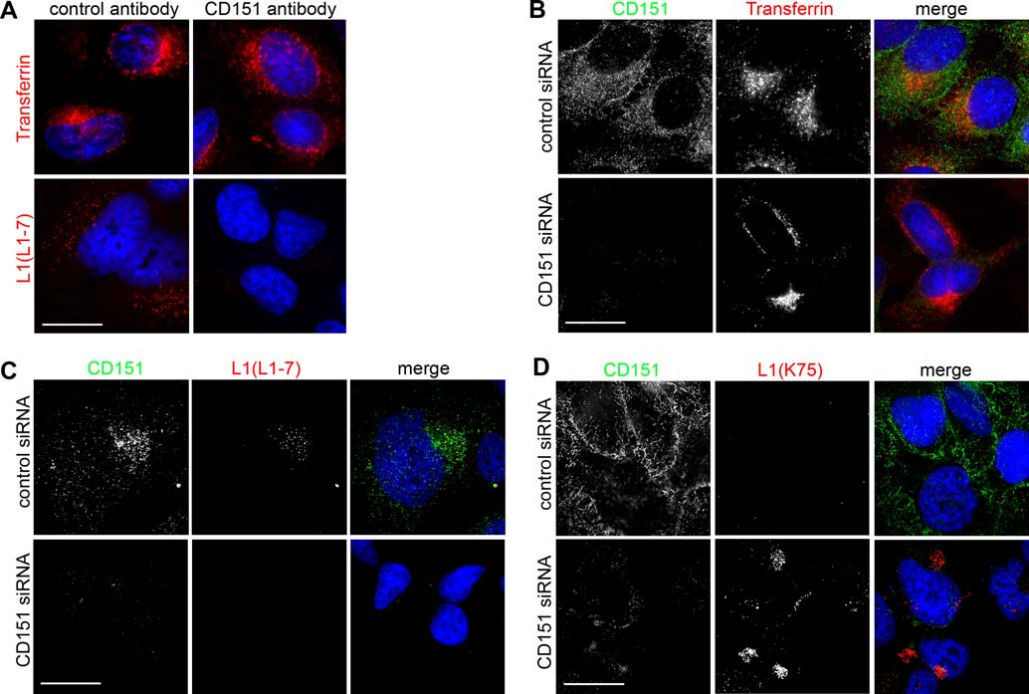
Inhibition of HPV16 pseudovirus entry in HeLa cells. (A) HeLa cells were treated with control and CD151 specific antibody as indicated. Entry of AlexaFluor conjugated transferrin (upper panels) or HPV16 PsVs (lower panels) was analyzed. Cells were fixed with MetOH and stained with monoclonal L1 (L1-7) antibody. (B) HeLa cells were transfected with control (upper panels) or CD151 siRNA (lower panels). Entry of AlexaFluor conjugated transferrin was analyzed. (C, D) HeLa cells were treated with siRNA and infected with HPV16 PsVs for 12 hours. Cells were either fixed and permeabilized with methanol and PsV uptake was analyzed by immunostaining with monoclonal L1 (L1-7, red) and polyclonal anti-CD151 antibodies (green) (C), or cells were fixed with paraformaldehyde and surface staining was performed with polyclonal L1 antibody (K75, red) and monoclonal anti-CD151 (green) antibody as indicated (D). Bars 20 µm.

We additionally analyzed the importance of CD151 for entry of HPV16 PsVs in HeLa cells using immunofluorescence studies. Again, cells were treated with control or CD151 specific antibodies prior to infection. Strikingly, antibody treatment had no effect on endosomal uptake of transferrin (Figure 8A, upper panels), whereas entry of virions was clearly inhibited (lower panels). Similarly, the effect of CD151 depletion by siRNA treatment on PsV entry was investigated. CD151 knockdown led to an almost complete loss of the tetraspanin-specific signal (Figure 8B, first column). However, uptake of transferrin was not affected (Figure 8B, second column). In contrast, entry of virions was completely blocked in CD151 depleted cells (Figure 8C). We hypothesized that depletion of CD151 might block viral entry at the plasma membrane. Therefore we analyzed HPV16 localization in CD151 siRNA-treated cells. As shown in Figure 8C (and Figure 1), 12 hours after infection we no longer detected viral particles on the cell surface in untreated cells reflecting complete uptake of PsVs (Figure 8C, control siRNA, L1(K75). Notably, in CD151-depleted cells uptake of PsVs was inhibited and PsVs remained on the cell surface (Figure 8C, CD151 siRNA, L1(K75)). Altogether, these results suggested that tetraspanins on the plasma membrane of epithelial cells are important for a clathrin- and caveolin-independent entry process of HPV16.

## Discussion

For many viruses, endocytic entry into their host cells occurs in a series of tightly controlled, consecutive steps involving binding to the cell surface, lateral diffusion, signaling, and internalization. Viral uptake often takes place via clathrin- or caveolar/raft-mediated endocytosis. In the case of HPV16 it was reported that entry is mediated by clathrin-dependent endocytosis [12], [40]. This conclusion was based on the inhibition of infection by the drug chlorpromazine. As it is known that chlorpromazine exerts multiple effects on treated cells [27] we thought to investigate HPV16 entry using alternative, more specific approaches. In order to clarify the involvement of defined cellular endocytosis mechanisms we specifically inhibited clathrin- and caveolin-mediated pathways by two independent methods. First, we used the expression of dominant-negative mutants to block endogenous protein functions and second, we specifically depleted key functional molecules within these pathways by siRNA. Thereby, we were able to control possible side effects of each approach. Since it has recently been shown that papillomaviruses could be internalized without inducing productive infection [7], we also used two different methods to monitor HPV16 invasion. In each case we investigated uptake of viral particles in immunofluorescence studies and quantified infectivity of HPV16 PsVs. This strategy allowed us to correlate endocytosis with infection that is important for differentiation between productive and non-productive uptake of viral particles.

Using this setup of experiments, we first asked whether HPV16 entry and infection is dependent on clathrin-mediated endocytosis. We used dn mutants of eps15 that have been shown to block AP-2 function and clathrin-coated pit assembly [28]. Although AP-2 is the key adaptor complex regulating clathrin-mediated endocytosis, there might exist AP-2 independent clathrin-mediated uptake pathways [41]. Therefore, we also employed clathrin-heavy chain knockdown. Both approaches led to the same conclusion: productive endocytosis of HPV16 occurs clathrin-independent.

One well-defined clathrin-independent pathway is endocytosis mediated by caveolae. Several studies showed that HPV31, in contrast to HPV16 and HPV58, might use caveolin-dependent uptake for infection, implying that different HPV types use different entry mechanisms [12], [40]. Our data, using dominant negative mutants and siRNA-mediated depletion of caveolin-1, supported the results that have thus far been published indicating that HPV16 entry does not involve caveolae. Furthermore, the observation that MßCD did not inhibit infection suggested that lipid rafts are also not involved. Our results are in line with the notion that different papillomavirus types may use different entry routes and point to the possibility that HPV16 uses a novel pathway to enter cells for infection. Of note, simultaneous inhibition of clathrin- and caveolin-mediated endocytosis by siRNA led to an increase of infectivity. This phenomenon might be due to cross-regulation of different endocytic pathways. A change in the activity of a particular endocytic mechanism could be compensated by alterations in other pathways. It has been shown for example that inhibition of receptor-mediated endocytosis resulted in up-regulation of clathrin-independent fluid-phase endocytosis [42]. Therefore, increased infectivity of HPV16 in cells with inhibited clathrin- and caveolin pathways additionally argues for usage of an alternative endocytic mechanism. This conclusion is supported by our observation that entry of HPV16 is also independent of the GTP-binding protein dynamin. Dynamin was initially thought to function mainly in internalization of clathrin-coated vesicles [35], [37] but is now also known to control clathrin-independent uptake of caveolae [43] and other poorly described pathways [44]. Again, we detected increased infectivity of HPV16 when dynamin was depleted or its function was blocked.

In recent years there is increasing evidence for additional and alternative clathrin-, caveolin-, and dynamin-independent entry pathways [44], [45]. They are poorly characterized and it is not known whether specific microdomains of the plasma membrane are involved in these novel entry routes. In our study we observed that virions become associated with several tetraspanins including CD63 and CD151 on the cell surface during the invasion process. One important feature of the tetraspanin proteins is their capacity to laterally interact among each other leading to the formation of TEMs. Accordingly, we detected strong colocalization of CD63 and CD151 in the plasma membrane of HeLa cells. The number of virions that were found to be associated with tetraspanin microdomains on the cell surface increased, as the infection process progressed. Importantly, inhibition of HPV16 entry and infection by tetraspanin-specific antibodies and siRNA suggested that TEMs could act as platforms for clathrin-, caveolin-, and dynamin-independent virion entry.

There is increasing evidence that TEMs may be involved in the infection process of various viruses. The tetraspanin CD81 has been identified as interaction partner of the HCV envelope glycoprotein E2 [46] and anti-CD81 mAbs, as well as a recombinant, soluble form of the large extracellular domain of CD81 inhibited the entry of HCV into hepatoma cell lines [47], [48]. Similarly, it has been suggested that the tetraspanin CD63 plays a role in the entry process of HIV-1 as it has recently been reported that HIV-1 infection was inhibited by anti-CD63 antibodies and also by recombinant soluble forms of the large extracellular domain of human tetraspanins [20], [21]. We were able to inhibit HPV16 infection by treatment of the cells with antibodies or siRNAs against CD63 and CD151. Their capacity to interact with other membrane components and to assemble into microdomains on the plasma membrane enables these molecules to serve as recipients of cargoes from primary receptors, like HSPGs. Binding of cargo to these functional platforms may then trigger endocytic uptake processes.

Which mechanisms are used for endocytosis of tetraspanins from the plasma membrane is not clear. A recent study found that syntenin-1, a component of TEMs, binds to the cytoplasmic tail of CD63 and could mediate a slow, clathrin-independent endocytosis of CD63 [49]. In addition, it was reported that CD151 is internalized via a dynamin-independent but actin-dependent endocytic pathway [50]. HPV16 might act as an important tool to clarify mechanisms and factors involved in uptake of tetraspanins and TEM-associated proteins.

Antibodies or siRNA targeting CD151 exerted the strongest inhibitory effect on HPV16 infection. CD151 is highly expressed in epithelial cells of the basal layer that are the target cells of HVP infection. Characteristically, CD151 is present on cells juxtaposed with basement membranes and is localized predominantly on the cell surface in contact with this membrane [51]. Furthermore, it is a component of hemidesmosomes, which mediate attachment of epithelial cells [38]. These observations together with the data presented in our study suggest that TEMs that are enriched in CD151 may indeed serve as entry platforms for HPV16 *in vivo*. Regarding the inhibitory mechanisms of the antibodies, we have preliminary data showing that treatment of the cells with tetraspanin-specific antibodies results in increased uptake of TEM proteins. Thereby, entry platforms of HPV16 may be depleted on the cell surface. Similarly, knockdown of tetraspanins by specific siRNAs may result in reorganization of TEM platforms that are no longer functional for virus entry. Regarding this aspect, we found that depletion of CD63 does not affect the amount of CD151 on the cell surface and vice versa (data not shown). This would support the notion that a specific TEM-organization is essential for mediating HPV16 uptake.

In previous studies, α6-integrin was proposed as a candidate receptor for HPV16 [9], [10]. We also detected reduction of infection with an antibody against α6-integrin (data not shown). It should be noted that CD151 is a prime interaction partner for integrin α6β4 in keratinocytes [38]. Therefore, it is conceivable that surface level depletion of CD151 by siRNA might induce a simultaneous decrease of its interaction partner α6β4, a possible important target for HPV16 invasion. However, we and others found that knock down of CD151 in human cells does not influence the surface level of partner integrins but disrupted their association with TEMs (data not shown) [52]. This implies that α6-integrin does not act inherently as the receptor for HPV16. It rather suggests that the specific environment of TEMs is a key feature for efficient infection of HPV16.

In summary, our data indicate that, following binding of HPV16 to the cell surface, virions specifically associate with microdomains containing the tetraspanins CD63 and CD151. We propose that this is important for the formation of specialized platforms for their uptake. This is based on the observation that tetraspanin-specific antibodies and siRNA inhibited HPV16 uptake and infection. Importantly, endocytosis of virions occurs by a process that is independent of clathrin- and caveolin-mediated mechanisms. Investigations into the signaling processes that are possibly triggered by binding of virions to tetraspanin microdomains are obviously called for. Delineation of these events will enhance our understanding of the mechanisms underlying infection by HPV16 and those agents that may utilize a TEM-specific entry route. These possibly include HIV and HCV.

## Materials and Methods

### Cell lines and pseudovirions

The human embryonic kidney cell line 293TT was obtained from Chris Buck [23]. The human cervix carcinoma cell line HeLa was purchased from the German Resource Centre for Biological Material (DSMZ). All cell lines were grown at 37°C in DMEM supplemented with 10% FCS, 1% Glutamax I, 1% modified Eagle medium nonessential amino acids and antibiotics.

HPV16 pseudovirions were prepared as previously described [23], [53]. Briefly, expression plasmids carrying codon-optimized HPV16 L1 and L2 cDNA [54] were co-transfected with a marker plasmid coding for GFP_2_-NLS, EYFP or DsRed1 into 293TT cells. 48 hours post-transfection, the cells were processed to lysis and nuclease digestion. The pseudovirions were purified from the cell lysates by Optiprep gradient centrifugation [23].

### Antibodies

The HPV16 L1-specific antibodies mAb L1-7 (mouse monoclonal) and K75 (rabbit polyclonal) have been described previously [25], [55]. Mouse monoclonal antibodies anti-clathrin heavy chain (CHC, clone 23), and anti-CD63 (H5C6) were obtained from BD Biosciences and the mouse anti-α-Tubulin antibody was from Sigma (B-5-1-2). Monoclonal mouse anti-CD151 (clone 11G5a) and anti-CD63 (sc-5275) were obtained from Serotec and Santa Cruz Biotechnologies, respectively. Rabbit anti-Caveolin1 (ab18199) was purchased from Abcam. Rabbit polyclonal anti-CD81 (sc-9158) and CD151 (sc-33123) antibodies, as well as goat anti-Dynamin-2 (sc-6400) antibody were obtained from Santa Cruz Biotechnologies.

### Plasmids

The Eps15 mutants: DIIIΔ2 (control), DIII and EH29 (EΔ95/295) all subcloned in pEGFP-C2 were kind gifts from Alexandre Benmerah (Université Paris, Paris, France) [28] and the plasmids for GFP tagged Caveolin1 (GFP-Cav, Cav-GFP) were provided by Hüseyin Sirma (Heinrich-Pette-Institut, Hamburg, Germany). The GFP tagged dynamin-2 mutant K44A (Dyn2K44A-GFP) was kindly provided by Sandra Schmid (The Scripps Research Institute). Codon-optimized HPV16 L1 and L2 expression plasmids were obtained from Martin Müller [54]. The GFP2-NLS marker plasmid has been described previously [56]. The marker plasmid pEYFP-C1 and pDsRed1-C1 were purchased from CLONTECH.

### siRNA Experiments

The clathrin heavy chain targeting siRNAs CHC 1 (AACCUGCGGUCUGGAGUCAAC·TT) [57] and CHC 2 (Hs_CLTC_10HP) were purchased from Sigma and Qiagen, respectively. The CD151 #1 (CAUGUGGCACCGUUUGCCU·TT) [58] and CD151 #2 (GCAGGUCUUUGGCAUGA TT) [59] specific siRNAs were obtained from Sigma. The caveolin-1 siRNA (Hs_CAV1_6), dynamin-2 siRNA (GW VAL siRNA Hs_DNM2_8, SI2654687), and the nonsilencing control siRNA (AllStars Neg. Control siRNA) were obtained from Qiagen. HeLa or 293TT cells were transfected with 30 nM of siRNA using Lipofectamine RNAiMAX according to manufacturer's cell line optimized instructions. Subsequent experiments were done 48 hours after siRNA transfection. Knockdown efficiencies were quantified at protein level by Western blot or flow cytometry.

### Transferrin and Cholera toxin uptake

HeLa cells were incubated with 35 µg/ml human transferrin labeled with AlexaFluor546 (Molecular Probes) or 0.5 µg/ml Cholera toxin subunit B (CtxB) labeled with AlexaFluor594 (Molecular Probes) for 50 min or 30 min, respectively. Remaining transferrin on the cell surface was removed by treatment with acid-wash solution (260 mM citric acid, 125 mM Na_2_HPO_4_) for 1 min. Labeled cells were finally washed twice with cold PBS, fixed in methanol and processed for immunofluorescence.

### Pseudovirus infection assay

293TT or HeLa cells grown in 24-well or 96-well plates were infected with approx. 100 p/cell HPV16 pseudovirions from OptiPrep gradients. After 48–72 hours incubation at 37°C, infection events reflected by fluorescent cells (expressing the marker plasmid) were determined by surveying the whole well in a fluorescence microscope and/or by flow cytometry (min. 5×10^4^ cells acquired).

Antibody infection inhibition assays were done by preincubating cells with 30 µg/ml of the indicated antibodies for 1 hour prior to infection.

### Immunofluorescence

HeLa cells were grown on coverslips. After transfection and/or infection (approx. 100 p/cell), cells were fixed with methanol (−20°C, 5 min) or 2% paraformaldehyde (PFA)/PBS (4°C, 10 min). For dynasore inhibition cells were pretreated for 30 minutes at 37°C with 80 µm dynasore in DMEM or 0,8% DMSO only (control). Dynasore was added during the whole infection process. Fixed cells were washed three times with 1% BSA/PBS and blocked 10 min in 1% BSA/PBS. Coverslips were incubated for 1 h at 37°C with the indicated antibodies. After washing with BSA/PBS, coverslips were again blocked for 10 min with 1% BSA/PBS and subsequently incubated at 37°C with Alexa-conjugated specific secondary antibodies (Invitrogen) for 45 min. DNA was stained with Hoechst 33342 (Sigma) and is shown in blue. Coverslips were washed with BSA/PBS and PBS and mounted onto slides using Fluoprep mounting medium (bioMérieux). Images were acquired using a Zeiss Axiovert 200 M microscope equipped with a Plan-Apochromat 100× (1.4 NA) and a Zeiss Axiocam digital camera. Axiovision software 4.6 was used for merging pictures. Images were deconvoluted using the software supplied by Zeiss (Axiovision 4.6). Tiffs were assembled into figures using Photoshop CS2 (Adobe). Quantification of colocalization was done using the colocalization module of Axiovision 4.6 (Zeiss). PsVs on the surface of a given cell were counted and the colocalizing fraction was determined. For each time point, five individual cells were analyzed.

### Electron microscopy

HeLa cells were grown on a 50 µm thick, gas-permeable lumox™ film in a 96 well lumox™ plate (greiner bio-one). Cells were exposed to HPV16 PsVs (approx. 500 p/cell) for 4 hours and fixed with 2.5% paraformaldehyde for 15 min at room temperature. Cells were washed three times with PBS, 1% BSA and blocked for 30 minutes at room temperature with PBS, 1% BSA. Cells were immunostained with mouse anti-CD151 monoclonal antibody (clone 11G5a, Serotec) followed by 10 nm gold-conjugated goat anti-mouse secondary antibody (British Biocell). Infected control cells were labeled with 10 nm gold conjugated anti-mouse secondary antibody only. Monolayer of cells was postfixed with 2.5% glutaraldehyde in PBS for 45 minutes at room temperature and embedded in Epon 812 according to standard protocols directly on the film base. 70 nm ultrathin sections were cut, stained with 1% lead citrate and 2% uranyl acetate and finally analyzed in Zeiss EM 902 electron microscope, equipped with TRS digital camera.

## Supporting Information

Figure S1 Cells fixed with paraformaldehyde (PFA) do not show intracellular labeling. HeLa cells were either fixed with 2% PFA or Methanol (MetOH) and immunostained with a mouse anti-α-tubulin antibody and an AlexaFluor-conjugated secondary antibody. PFA-fixation resulted in non-permeabilized cells showing no intracellular staining. Bar, 20 µm.
(0.13 MB PDF)

Figure S2 Detection of extra- and intracellular pseudovirions. HeLa cells were incubated with HPV16 PsVs for 8 hours. Cells were fixed with 2% PFA and the plasma membrane was either left intact (non-permeabilized) or permeabilized with 0,2% Triton X-100 (PFA+Triton) or methanol (MetOH). For immunofluorescence co-staining of the PsVs a rabbit polyclonal antiserum (K75) together with the mouse monoclonal L1-7 antibody and AlexaFluor-conjugated secondary antibodies were used. The L1-7 antibody only detects K75-positive particles in intracellular compartments. Bar, 20 µm.
(0.18 MB PDF)

Figure S3 Clathrin independent entry and infection of HPV16. 293TT cells were transfected with GFP-tagged dominant-negative Eps15-mutant (GFP-DIII), inhibitor of clathrin-mediated endocytosis, or a control (GFP-DIIIdelta2) for 24 hours and then incubated with PsVs. 48 hours post infection cells were analyzed by immunofluorescence microscopy. Infected cells show expression of the DsRed marker plasmid. Bar in C, 100 µm.
(0.56 MB PDF)

Figure S4 Selective detection of CD63 on the cell surface and in intracellular compartments. HeLa cells were fixed with 2% paraformaldehyde (PFA) to leave the plasma membrane intact or fixed and permeabilized with methanol (MetOH) and stained with anti-CD63 antibody (green). Images were captured in Z series using deconvolution fluorescence microscopy. Top section or middle sections are shown as indicated. Depending on the focusing plane, in PFA fixed cells CD63 was detected on the whole cell surface (top section) or at the cell borders (middle section). In MetOH fixed cells surface staining of CD63 was lost and only intracellular compartments were detected. Bars 20 µm.
(0.49 MB PDF)
