# Supplementary figures and images for: Clathrin- and Caveolin-Independent Entry of Human Papillomavirus Type 16—Involvement of Tetraspanin-Enriched Microdomains (TEMs)
Source: PLoS One. 2008 Oct 2;3(10):e3313. doi: 10.1371/journal.pone.0003313 (PMC2561052; doi:10.1371/journal.pone.0003313)

Figure S1

PFA

tubulin

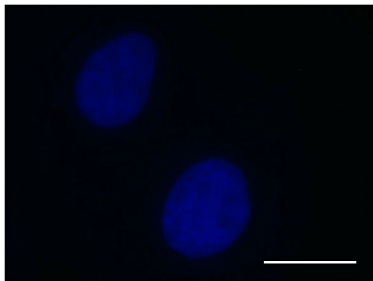

MetOH

tubulin

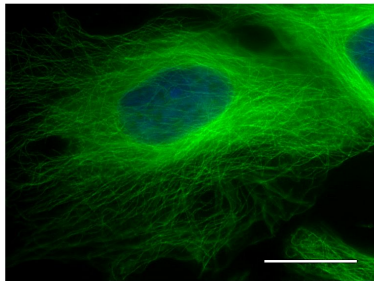

Supplement: Figure S1 — Cells fixed with paraformaldehyde (PFA) do not show intracellular labeling. HeLa cells were either fixed with 2% PFA or Methanol (MetOH) and immunostained with a mouse anti-α-tubulin antibody and an AlexaFluor-conjugated secondary antibody. PFA-fixation resulted in non-permeabilized cells showing no intracellular staining. Bar, 20 µm. (0.13 MB PDF) [file pone.0003313.s001.pdf]

Figure S2

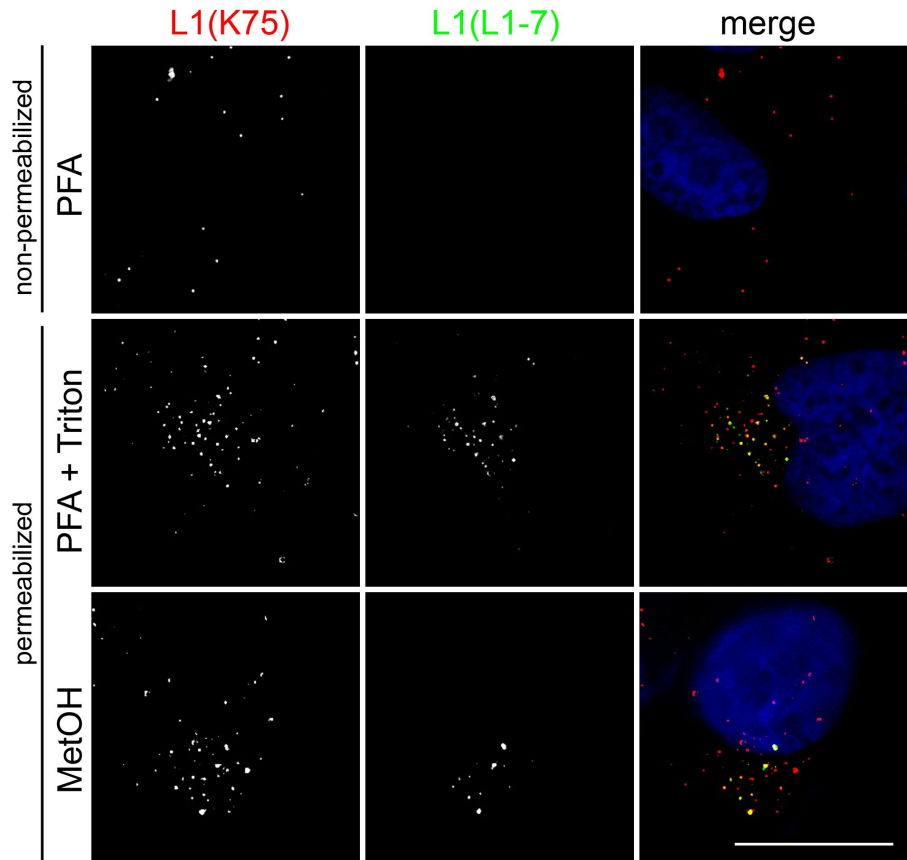

Supplement: Figure S2 — Detection of extra- and intracellular pseudovirions. HeLa cells were incubated with HPV16 PsVs for 8 hours. Cells were fixed with 2% PFA and the plasma membrane was either left intact (non-permeabilized) or permeabilized with 0,2% Triton X-100 (PFA+Triton) or methanol (MetOH). For immunofluorescence co-staining of the PsVs a rabbit polyclonal antiserum (K75) together with the mouse monoclonal L1-7 antibody and AlexaFluor-conjugated secondary antibodies were used. The L1-7 antibody only detects K75-positive particles in intracellular compartments. Bar, 20 µm. (0.18 MB PDF) [file pone.0003313.s002.pdf]

Figure S3

Eps15-GFP + DsRed

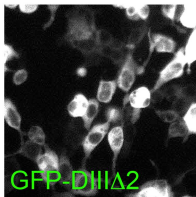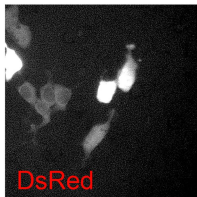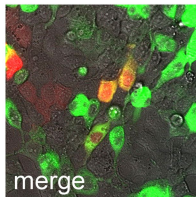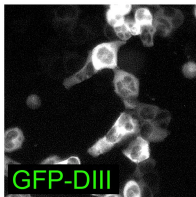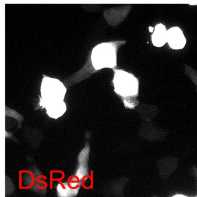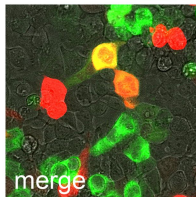

Supplement: Figure S3 — Clathrin independent entry and infection of HPV16. 293TT cells were transfected with GFP-tagged dominant-negative Eps15-mutant (GFP-DIII), inhibitor of clathrin-mediated endocytosis, or a control (GFP-DIIIdelta2) for 24 hours and then incubated with PsVs. 48 hours post infection cells were analyzed by immunofluorescence microscopy. Infected cells show expression of the DsRed marker plasmid. Bar in C, 100 µm. (0.56 MB PDF) [file pone.0003313.s003.pdf]

Figure S4

PFA (top section)

PFA (middle section)

MetOH (middle section)

CD63 + DNA

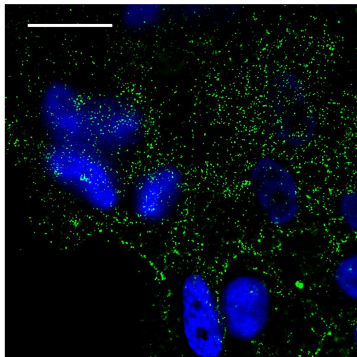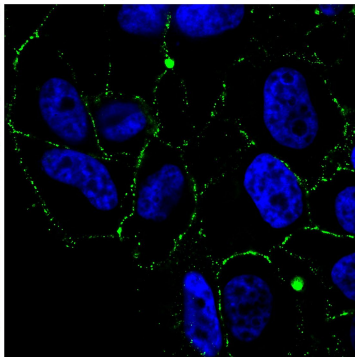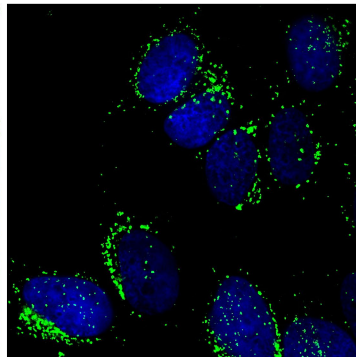

Supplement: Figure S4 — Selective detection of CD63 on the cell surface and in intracellular compartments. HeLa cells were fixed with 2% paraformaldehyde (PFA) to leave the plasma membrane intact or fixed and permeabilized with methanol (MetOH) and stained with anti-CD63 antibody (green). Images were captured in Z series using deconvolution fluorescence microscopy. Top section or middle sections are shown as indicated. Depending on the focusing plane, in PFA fixed cells CD63 was detected on the whole cell surface (top section) or at the cell borders (middle section). In MetOH fixed cells surface staining of CD63 was lost and only intracellular compartments were detected. Bars 20 µm. (0.49 MB PDF) [file pone.0003313.s004.pdf]
